# Supplementary material for: Naturido alleviates amyloid β1–42-induced adverse effects in a transgenic Caenorhabditis elegans model of Alzheimer’s disease
Source: PLoS One. 2025 Mar 26;20(3):e0320636. doi: 10.1371/journal.pone.0320636 (PMC11940425; doi:10.1371/journal.pone.0320636)
Supplement: Attachment — Submitted filename: Response to Reviewers piyamas PLOSONE.docx [file pone.0320636.s004.docx]

Dear Reviewer #1,

Thank you for your review comments on our manuscript (PONE-D-24-54123). Before responding point-by point, we would like to express our deep thanks for your constructive and thoughtful comments. We have taken these comments and suggestions into account in our revised manuscript as described below.

We hope that the revised manuscript is now suitable for publication in PLOS ONE.

*RE: Reviewer #1*

1) In the introduction the authors missed the citation to another work on an AD model of C.elegans involving the anti-AD effect of a natural protein, alpha-casein, due its idp and chaperone-like properties and its ability to sequester oligomeric intermediates in amyloid aggregation [Paterna et al. 2023, 10.1021/acschemneuro.3c00239].

Thank you for this suggestion. We are sorry that we missed this important study. According to this comment, we cited this study in the “Introduction” section.

2) In the description of Fig.1A, where the chemotaxix assay is described, is not clear if A and B are points or regions or even lines delimiting a circle of radius 2.5cm. Sorry, I could not make it to understand. Can you better explain?

We are very sorry that we did not describe this point explicitly. We revised Fig. 1 by highlighting the regions used for counting. Additionally, we renamed the region B to region C (for control), and revised the manuscript accordingly.

3) What does HT assay means? I missed acronym list.

We apologize for the lack of consistency in terminology. The HT assay refers to the 5-hydoroxytryptamine (serotonin) assay. In the revised manuscript, we consistently used the term “serotonin hypersensitivity assay” throughout the text.

4) Comment on the fact that also mocks dye after 40 days. Is it the worm natural lifespan?

Thank you for your constructive comments. The average lifespan of the control worms was approximately 30 days, with a maximum of 40 days. Although lifespan depends on environmental factors such as temperature and food quality, under our experimental conditions, the control worms consistently exhibited a lifespan within this range. We have added the description to the “Results” section.

5) Comment on the fact that under the treatment with N50 it seems that a tiny toxicity is present in the CL2122 lifespan (not clear to me if it is statistically consistent?), while improvements are noticed in the locomotion and survival experiments.

According to this comment, we performed a Wilcoxon test in addition to the log-rank test for the lifespan data, but neither test revealed statistically significant differences between CL2122 and CL2122 N50 results. We also conducted a comprehensive reassessment of the statistical analyses for the entire study (chemotaxis, serotonin hypersensitivity, and locomotion) and revised some descriptions and figures. Although no significant differences were observed in the serotonin hypersensitivity of the control worms, significant differences were observed in locomotion, and this has been noted in the revised text.

6) Remember to give reference of all the products used, with brand and product number, in the right Reagents Section.

We are very sorry that we did not describe this point explicitly. We have added the product number of donepezil hydrochloride, as well as the brand and product number of serotonin. For other reagents, we used commonly available products, and this point was described in the revised manuscript.

7) Some supplementary information, such as videos, could be helpful for the unexpert reader to have a representation of the main results of the study, like the rescue in body bending in the AD case for example?

Thank you for this suggestion. We recorded videos of several experiments and included them as supplementary materials in our revised manuscript.
